# Zerumbone, a tropical ginger sesquiterpene, ameliorates streptozotocin-induced diabetic nephropathy in rats by reducing the hyperglycemia-induced inflammatory response

**DOI:** 10.1186/1743-7075-10-64

**Published:** 2013-10-17

**Authors:** Thing-Fong Tzeng, Shorong-Shii Liou, Chia Ju Chang, I-Min Liu

**Affiliations:** 1 Department of Pharmacy & Graduate Institute of Pharmaceutical Technology, Tajen University, Yanpu Township, Pingtung County, Taiwan, R.O.C

## Abstract

**Background:**

Zerumbone is one of the pungent constituents of *Zingiber zerumbet* (L) Smith (Zingiberaceae family). The aim of the present study was to examine the effects of zerumbone in rats with streptozotocin-induced diabetic nephropathy (DN).

**Methods:**

Diabetic rats were treated orally with zerumbone (20 or 40 mg/kg/day) for 8 weeks. Changes in renal function-related parameters in plasma and urine were analyzed at the end of the study. Kidneys were isolated for pathology histology, immunohistochemistry, and Western blot analyses.

**Results:**

Diabetic rats exhibited renal dysfunction, as evidenced by reduced creatinine clearance, increased blood glucose, blood urea nitrogen and proteinuria, along with marked elevation in the ratio of kidney weight to body weight, that were reversed by zerumbone. Zerumbone treatment was found to markedly improve histological architecture in the diabetic kidney. Hyperglycemia induced p38 mitogen-activated protein kinase activation, leading to increased infiltration of macrophages and increased levels of interleukin (IL)-1, IL-6 and tumor necrosis factor-α. All of the above abnormalities were reversed by zerumbone treatment, which also decreased the expression of intercellular adhesion molecule-1, monocyte chemoattractant protein-1, transforming growth factor-β1 and fibronectin in the diabetic kidneys.

**Conclusions:**

The beneficial effect of zerumbone in rats with DN is at least in part through antihyperglycemia which was accompanied by inhibition of macrophage infiltration via reducing p38 mediated inflammatory response.

## Background

Diabetic nephropathy (DN) is a major cause of end-stage renal failure worldwide. DN has not been traditionally considered an inflammatory disease, however, recent studies have shown that kidney inflammation is crucial in promoting the development and progression of DN [1,2]. Many studies have demonstrated that macrophage infiltration into glomeruli is associated with the progression of DN [3]. Monocyte chemotactic protein-1 (MCP-1) and intercellular adhesion molecule-1 (ICAM-1), which make the monocytes/macrophages extravasculate from the blood-stream and attract to the kidney tissue, promote the development of DN [4,5]. The inflammatory cytokines such as tumor necrosis factor-α (TNF-α), interleukin (IL)-6 and IL-1β also play an important role in the development of renal hypertrophy and hyperfunction during the development of DN [6]. Therefore, it may be a novel therapy strategy for DN to reduce the infiltration of inflammatory cells.

*Zingiber zerumbet* Smith is one kind of plant growing mainly in Southeast Asia, which has been demonstrated to possess antinociceptive, anti-inflammatory, antiulcer, antihyperglycemic, and antiplatelet activities [7]. As a major compound extract, zerumbone (2,6,9,9- tetramethyl-[2*E*,6*E*,10*E*]-cycloundeca-2,6,10-trien-1-one) is currently explored for its potential broad use on cancers, leukemia, as well as virus infection [8]. Several studies revealed that zerumbone can inhibit tumor initiation and proliferation. This compound inhibits the proliferation of colon and breast cancers, with minimal effects on normal cells [9]. Zerumbone was also shown to suppress skin tumor initiation and promotion, inhibit inducible nitric oxide synthase and cyclooxygenase-2 expression, suppress free radical generation, and inhibit TNF-α release in activated leukocytes [10]. The ethanol extract of *Z. zerumbet* be beneficial in ameliorating diabetic renal damage has been previously explored [11]. However, there are no scientifically proven data to show zerumbone is the components of *Z. zerumbet* responsible for the protective effect on diabetic renal tissues. Thus, we investigated the effect of zerumbone on macrophages accumulation and the expression of ICAM-1, MCP-1 and proinflammatory cytokines in experimental diabetic kidney.

Accumulating evidence also suggested the development of DN was associated with the activation of several signal pathways, including mitogen-activated protein kinase (MAPK) signaling cascade. p38 MAPK (p38) participates in the intracellular signal transduction and production of cytokines and chemokines [12]. In diabetic animal models, p38 activity rapidly increases in glomeruli and tubules after the induction of hyperglycaemia, and is also found in the accumulating kidney interstitial cells associated with advanced nephropathy. The posibility of zerumbone to prevent the elevated renal p38 activity in diabetic rats and thereby attenuated the pathological abnormalities of DN was also elucidated in the present study.

## Methods

### Animal models

Male Wistar rats (8 to 10 weeks of age, 200–250 g) were obtained from the Animal Center of National Cheng Kung University Medical College. To induce diabetes rats were given a single intravenous injection of 60 mg/kg streptozotocin (STZ; Sigma-Aldrich, Inc., St. Louis, Mo., USA). Animals were considered to be diabetic if they had plasma glucose concentrations of 350 mg/dL or greater, in addition to polyuria and other diabetic features. All studies were carried out two weeks after the injection of STZ. All animal procedures were performed according to the Guidelines for the Care and Use of Laboratory Animals of the National Institutes of Health (United States), as well as the guidelines of the Animal Welfare Act. The study was conducted with the approval of the Institutional Animal Care and Use Committee (IACUC) at Tajen University (approval number: IACUC 99–24; approval date: December 23, 2011).

### Treatment protocols

STZ-diabetic rats in the treatment group were dosed with 20 or 40 mg/kg zerumbone (≥ 98%; Sigma-Aldrich, Inc.) in distilled water (1.5 mL/kg) by oral gavage once daily for eight weeks. The dosage regime was selected based on a previous report demonstrating that zerumbone at 40 mg/kg was potentially effective in improving acute necrotizing pancreatitis in rat [13]. Another group of STZ-diabetic rats was treated orally for eight weeks with 5 mg/kg/day rosiglitazone (purity ≥ 99.0%, Sigma-Aldrich, Inc.). The dose of rosiglitazone was based on studies with long-term treatment in STZ-diabetic rats [14]. A vehicle-treated groups of STZ-diabetic rats and normal rats were give 1.5 mL/kg distilled water by oral gavage over the same period. Animals had free access to standard rat diet (Harlan Teklad, Madison, WI, USA; Cat. No. 2018) and water throughout the entire treatment period. At the end of the eight-week treatment, the rats were weighed, and blood samples were collected from a tail vein. Treatment was continued even though the plasma glucose of STZ-diabetic rats was lower than 350 mg/dL during the eight-week treatment period. The evening prior to blood sample collection, animals were restricted to 3 g of chow (given at 18:00 h), which was consumed immediately, and thereafter had access to only water. The animals were transferred to metabolic cages (Shineteh Instruments Co., Ltd, Taipei, Taiwan), and urine was collected for 24 hours under a layer of toluene (to inhibit bacteria growth) and stored at 4°C for later analysis. Toluene had no detectable effect on the estimation of albumin and creatinine in the urine samples. Following urine collection, rats were sacrificed using an intraperitoneal injection of sodium pentobarbital (50 mg/kg).

The kidneys were dissected and rinsed with cold isotonic saline and weighed. The right kidney were stored immediately at -80°C in liquid nitrogen for biochemical determinations and Western blot analyses. Other kidney tissues were fixed in 10% neutralized formalin for histology.

### Blood sampling and analysis

Blood samples were centrifuged at 2 000 × g for 10 minutes at 4°C, and plasma was divided into aliquots for subsequent analyses. Plasma glucose concentration was determined using a diagnostic kit from BioSystem (Barcelona, Spain; Cat. No. COD12503). Serum creatinine (Scr) concentration was determined using a commercial assay kit purchased from Diagnostic Chemicals Limited (Connecticut, USA; Cat. No. 221–30). Blood urea nitrogen (BUN) was determined by kinetic reagent (Diagnostic Chemicals Limited, Cat. No. 283–30). Commercial enzyme-linked immunosorbent assay (ELISA) kits were used to quantify glycosylated hemoglobin (HbA_1c_) levels (Integrated Bio Ltd., Taipei, Taiwan; Cat. No. CSB-E08140r). The rat insulin ELISA kit was obtained from LINCO Research, Inc. (St. Charles, MO, USA; Cat. #EZRMI-13 K). All analyses were performed in accordance with the instructions provided by the manufacturers.

### Analysis of urine parameters

The 24-hour urine samples collected from each diabetic rat and age-matched control was centrifuged at 2 000 × g for 10 minutes. Urinary albumin concentrations were measured with the Nephrat II ELISA kit (Exocell, PA, USA; Cat. No. NR002). The concentration of creatinine in pooled urine samples was determined using a commercial assay kit (Diagnostic Chemicals Limited; Cat. No. 221–30). All analyses were performed in accordance with the manufacturer’s instructions. Creatinine clearance (Ccr) was calculated in individual rats using the relationship: Ccr = urine creatinine × (urine volume/plasma creatinine) × time [15].

### Renal cytokines determination

Renal tissue was homogenized in 10 mmol/L Tris–HCl buffered solution (pH 7.4) containing 2 mol/L NaCl, 1 mmol/L EDTA, 0.01% Tween 80, 1 mmol/L PMSF, and centrifuged at 9 000 × *g* for 30 min at 4°C [16]. The resultant supernatant was used for cytokine determination. ELISA kits for the determination of TNFα (Cat. No. ab46070), IL-6 (Cat. No. ab100772), and IL-1β (Cat. No. ab100768) were obtained from Abcam Inc. (Cambridge, MA, USA). Samples were assayed in duplicates according to manufacturer’s instructions. The protein concentrations of kidney filtrate were determined using a Bio-Rad protein assay kit (Bio-Rad Laboratories, Japan) and bovine serum albumin (BSA) as a standard.

### Renal histological analysis

For histological analysis, the kidney was removed and embedded in paraffin to prepare 4-μm tissue slices. The tissue slices were stained with periodic acid-Schiff (PAS). The mesangial expansion index was scored in four levels from 0 to 3, with the index scores defined as follows [17]: 0, normal glomeruli; 1, matrix expansion occurred in up to 50% of a glomerulus; 2, matrix expansion occurred in 50 to 75% of a glomerulus; 3, matrix expansion occurred in 75 to 100% of a glomerulus. Scores were assigned for at least 30 glomeruli fromkidney slices fromeach animal, and the means were calculated. Each slide was scored by apathologist who was unaware of the experimental details.

### Immunohistochemistry

Formalin-fixed, paraffin-embedded kidney tissue sections were used for immunohistochemical staining. After deparaffinization and hydration, the slides were washed in Tris-buffered saline (TBS; 10 mmol/L Tris HCl, 0.85% NaCl, pH 7.2). Endogenous peroxidase activity was quenched by incubating the slides in methanol and 0.3% H_2_O_2_ in methanol. After overnight incubation with mouse monoclonal anti-rat monocyte/macrophage antibody (anti-ED-1) (Santa Cruz Biotechnology Inc. CA, USA; Cat. No. sc-59103) at 4°C, the slides were washed in TBS. Horseradish peroxidase-conjugated goat anti-mouse secondary antibody was then added, and the slides were incubated at room temperature for an additional 1 hour. The slides were washed in TBS, incubated with diaminobenzidine tetrahydrochloride as the substrate, and counterstained with hematoxylin. A negative control without primary antibody was included in the experiment to verify antibody specificity. Intraglomerular ED-1-positive cells were counted in 200 glomeruli/group at 400 × magnification.

### Western blotting

Protein extraction of isolated kidney was performed as follows [18]. The sample was homogenized in ice-cold in 1 mL of hypotonic buffer A [10 mmol/L HEPES (pH 7.8), 10 mmol/L KCl, 2 mmol/L MgCl_2_, 1 mmol/L DTT, 0.1 mmol/L EDTA, 0.1 mmol/L phenylmethylsulfonylfluoride]. A solution of 80 μL of 10% Nonidet P-40 was added to the homogenates, and the mixture was centrifuged for 2 min at 14 000 × g. The supernatant was collected as a cytosolic fraction for the assays of ICAM-1, MCP-1, fibronectin and transforming growth factor (TGF)-β1. The supernatant containing nuclear proteins was collected for p38 and phosphorylated p38 (pThr180/Tyr182)(P-p38).

Before immunoblotting, and the protein concentration of each tissue was determined using a Bio-Rad protein assay kit (Bio-Rad Laboratories, Japan) and BSA as a standard, to ensure equal loading among lanes. Cytosol (70 μg total protein) and nuclear extracts (50 μg total protein) were separated on a 7.5-15% polyacrilamide gel and electophoretically transferred to nitrocellulose membrane. Membranes were blocked with 5% non-fat dry milk in Tris-buffered saline Tween (20 mmol/L Tris, pH 7.6, 137 mmol/L NaCl, and 0.1% Tween 20) for 3 h at room temperature, followed by an overnight incubation at 4°C with polyclonal antibodies against rat ICAM-1 (Santa Cruz Biotechnology, Inc.; Cat. No. sc-1511), MCP-1 (Santa Cruz Biotechnology, Inc.; Cat. No. sc-1785), fibronectin (Abcam plc, Cambridge, UK, Cat. No. ab2413), TGF-β1(Santa Cruz Biotechnology, Inc.; Cat. No. sc-52893), p38 (Cell Signaling Technology; Cat. No. 9212), P-p38 (Cell Signaling Technology; Cat. No. 9211), or β-actin (Santa Cruz Biotechnology, Inc.; Cat. No. sc-130656). All antibodies were used at a dilution of 1:1000. Three times after washing with Tris-buffered saline Tween 20 (TBST), incubation with appropriate horseradish peroxidase-conjugated secondary antibodies were performed for 1 h at room temperature. After three additional TBST washes, the immunoreactive bands were visualized by enhanced chemiluminescence (Amersham Biosciences, Buckinghamshire, UK) according to the manufacturer’s instructions. Band densities were determined using ATTO Densitograph Software (ATTO Corporation, Tokyo, Japan) and quantified as the ratio to β-actin. The mean value for samples from the vehicle-treated normal rats on each immunoblot, expressed in densitometry units, was adjusted to a value of 1.0. All experimental sample values were then expressed relative to this adjusted mean value. Tissue sections were sampled from 4 independent experiments.

### Statistical analysis

The results are presented as mean ± standard deviation (SD). Statistical analyses were performed using one-way analysis of variance. Dunnett range post-hoc comparisons were used to determine the source of significant differences where appropriate. Renal histomorphology and morphologic analysis for PAS staining were analyzed statistically using the Kruskal-Wallis Test and Dunn’s Multiple Comparisons Test. Values of *p* < 0.05 were considered statistically significant.

## Results

### Effects of treatments on blood glucose levels

Eight weeks following the induction of diabetes, STZ-diabetic rats exhibited a significant increase in fasting blood glucose compared to rats in the normal control group. Treatment of STZ-diabetic rats for eight weeks with daily doses of zerumbone produced obvious blood glucose-lowering effects: 20 mg/kg, 18.9 ± 2.9% reduction; 40 mg/kg, 28.8 ± 3.1% reduction (Table 1). The mean HbA_1c_ level in STZ-diabetic rats was markedly higher than that in normal rats (Table 1). Daily treatment with zerumbone at doses of 20 and 40 mg/kg for eight weeks decreased the levels of HbA_1c_ in STZ-diabetic rats by 14.3 ± 3.2 and 23.1 ± 2.7%, respectively, relative to the values in vehicle-treated STZ-diabetic rats (Table 1). After 8 weeks of treatment with rosiglitazone, the plasma glucose concentrations and HbA_1c_ were approximately 32.3 ± 4.7 and 26.1 ± 3.8% lower in STZ-diabetic rats compared to their vehicle-counterparts (Table 1).

**Table 1:** Biochemical parameters in experimental animals at the end of the eight-week treatment

	**Normal rats**	**STZ-diabetic rats**			
	**Vehicle**	**Vehicle**	**ZER 20**	**ZER 40**	**RGZ**
Body weight (BW) (g/rat)	402.94 ± 16.88^d^	262.38 ± 16.14^b^	287.74 ± 15.92^b,c^	318.42 ± 17.64^a,c^	335.18 ± 18.10^a,d^
Kidney weight (KW) (g)	1.58 ± 0.32^d^	2.84 ± 0.22^b^	2.47 ± 0.35^b^	2.26 ± 0.27^a,c^	1.96 ± 0.29^d^
KW/BW ratio (%)	0.32 ± 0.06^d^	1.08 ± 0.11^b^	0.86 ± 0.07^b^	0.71 ± 0.08^b,c^	0.58 ± 0.03^a,d^
Plasma glucose (mg/dl)	95.72 ± 6.38^d^	445.02 ± 16.43^b^	361.72 ± 17.14^b,c^	317.48 ± 15.31^b,c^	301.62 ± 16.23^b,c^
HbA_1c_ (%)	4.88 ± 0.92^d^	14.57 ± 1.17^b^	12.48 ± 1.26^b^	11.21 ± 1.09^b^	10.76 ± 0.89^b,c^
Plasma insulin (μU/ml)	25.43 ± 0.51^d^	0.27 ± 0.14 ^b^	0.31 ± 0.12^b^	0.35 ± 0.19^b^	0.41 ± 0.21^b^
24-h urine volume (ml/day)	9.15 ± 2.14^d^	27.86 ± 3.17^b^	22.29 ± 3.08^b^	18.02 ± 3.72^a,c^	15.68 ± 2.83^a,c^
24-h urine protein (mg/day)	6.81 ± 3.26^d^	29.24 ± 5.71^b^	20.47 ± 4.82^b^	15.28 ± 3.62^a,d^	11.42 ± 4.74^a,d^
Scr (μmol/l)	35.13 ± 5.43^d^	87.87 ± 8.21^b^	72.89 ± 7.21^b^	64.14 ± 6.23^b,c^	58.24 ± 5.77^b,c^
BUN (mmol/l)	6.85 ± 1.32^c^	19.81 ± 1.84^b^	16.51 ± 1.75^b^	14.38 ± 1.76^b,c^	10.23 ± 1.56^a,c^
Ccr (ml/min)	4.26 ± 0.59^d^	1.84 ± 0.64^b^	2.21 ± 0.46^b^	3.02 ± 0.47^a,c^	3.46 ± 0.52^d^

STZ-diabetic rats were dosed by oral gavage once per day for eight weeks with 5 mg/kg/day rosiglitazone (RGZ), 20 mg/kg/day zerumbone (ZER 20), 40 mg/kg/day zerumbone (ZER 40). Normal or STZ-diabetic rats receiving vehicle treatment were given the same volume of vehicle (distilled water) used to dissolve test medications. Values (mean ± SD) were obtained for each group of 8 animals.

^a^*p* < 0.05 and ^b^*p* < 0.01 compared to the values of vehicle-treated normal rats.

^c^*p* < 0.05 and ^d^*p* < 0.01 compared to the values of vehicle-treated STZ-diabetic rats, respectively.

Insulin deficiency in these rats was shown by the finding that plasma insulin levels in STZ-diabetic rats were reduced to 0.27 ± 0.14 μU/ml following STZ injection, a level markedly lower than that of the normal rats (25.43 ± 0.51 μU/ml). Plasma insulin level in the STZ-diabetic rats was not modified by zerumbone or rosiglitazone repeatedly treatment because plasma insulin levels were 0.35 ± 0.19 and 0.41 ± 0.21 μU/ml in STZ-diabetic rats treated for eight weeks with zerumbone (40 mg/kg/day) or rosiglitazone.

### Effects of treatments on indices of renal function

STZ-diabetic rats showed an increase in 24-hour urine volume, accompanied by increase in urine protein excretion (Table 1). After eight weeks of zerumbone or rosiglitazone treatment, 24-hour urine volume and 24-hour urine protein excretion for STZ-diabetic rats were markedly less than those of their vehicle-treated counterparts (Table 1). In addition, Scr and BUN levels in STZ-diabetic rats were obviously higher than in rats from the normal control group. These levels were effectively reduced in STZ-diabetic rats treated for eight weeks with zerumbone relative to levels in their vehicle-counterparts (Table 1). In particular, increased Ccr in STZ-diabetic rats was obviously observed after eight weeks of zerumbone (40 mg/kg/day) or rosiglitazone treatment (Table 1).

### Effects of treatments on renal morphology

At the end of the eight-week treatment, the mean weight of the left kidney and the ratio of kidney weight to body weight in STZ-diabetic rats were 1.8 and 3.4 fold, respectively, relative to the values in the normal group (*p* < 0.01). Treatment of STZ-diabetic rats with 20 mg/kg/day zerumbone slightly reduced the the mean weight of the left kidney and the ratio of kidney weight to body weight, which were markedly reduced in STZ-diabetic rats treated for eight weeks with 40 mg/kg/day zerumbone or rosiglitazone (Table 1).

### Effects of treatments on kidney histopathology

**Figure 1:**
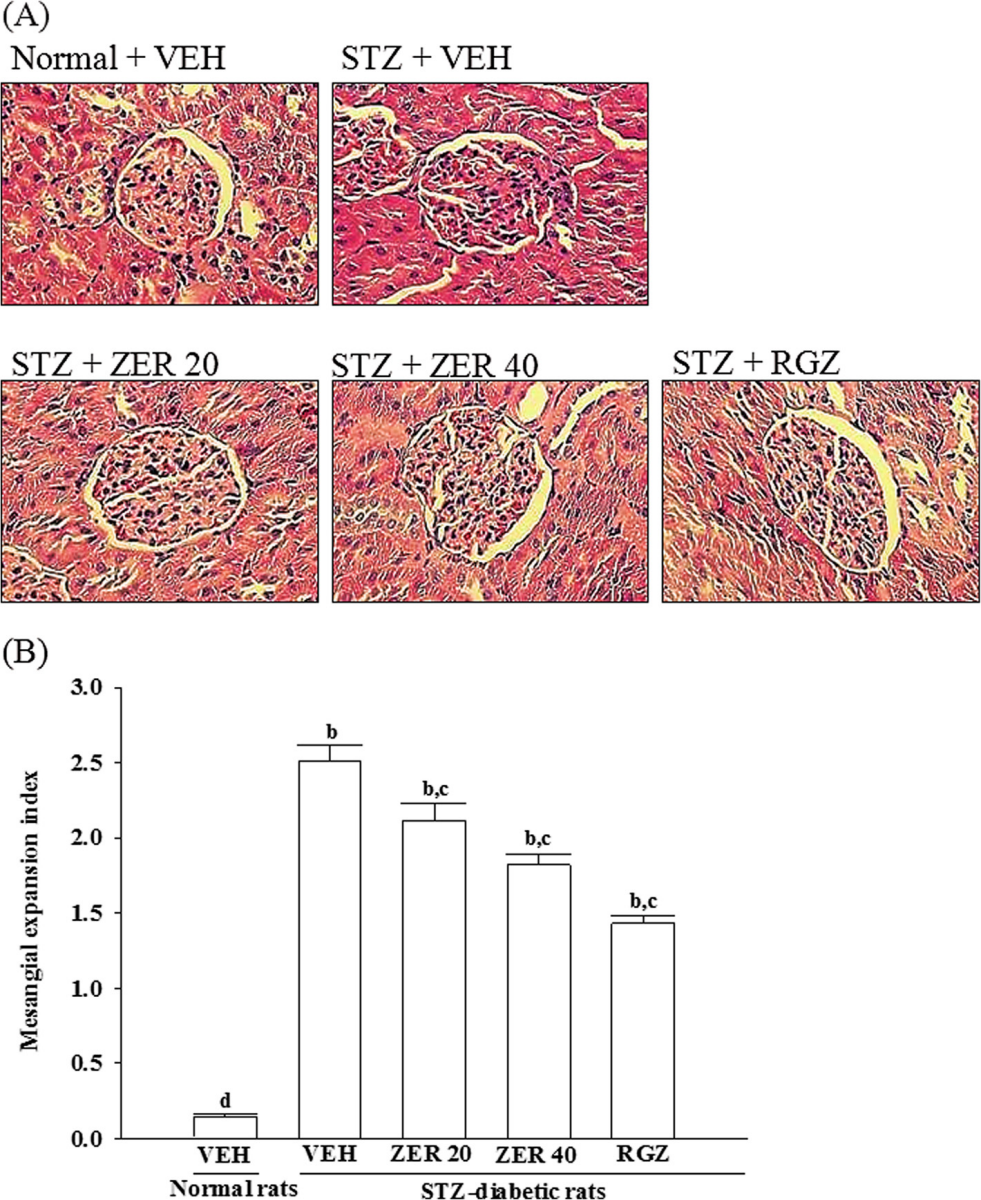
**Effects of treatments on kidney histopathology. (A)** Representative photomicrographs (original magnification, 400×) of PAS-stained kidney sections from STZ-diabetic rats treated for eight weeks with zerumbone (ZER) or rosiglitazone (RGZ). STZ-diabetic rats were dosed by oral gavage once daily for eight weeks with 20 mg/kg ZER (STZ + ZER 20), 40 mg/kg ZER (STZ + ZER 40) or 5 mg/kg RGZ (STZ + RGZ). Normal (normal + VEH) or STZ-diabetic rats receiving vehicle treatment (STZ + VEH) were administered the same volume of vehicle (VEH) used to dissolve test medications. **(B)** Results of quantification of the mesangial expansion index for each group. Values (mean ± SD) were obtained for each group of 4 animals. ^b^*p* < 0.01 compared to vehicle-treated normal rats. ^c^*p* < 0.05 and ^d^*p* < 0.01 compared to vehicle-treated STZ-diabetic rats, respectively.

The STZ-diabetic rats showed focal mesangial matrix expansion compared to normal control rats (Figure 1A). Quantification of renal pathology showed that mean mesangial area was significantly increased in diabetic rats, however, treatment with rosiglitazone for 8 weeks markedly ameliorated mesangial expansion when compared with the untreated STZ-diabetic rats (Figure 1B) After eight weeks of treatment, enlargement of the mesangia in glomeruli was mildly attenuated in the STZ-diabetic rats treated with 20 mg/kg/day of zerumbone. Quantitative analysis also showed that there was a marked decrease in the percentage of mesangial expansion in STZ-diabetic rats treated with 40 mg/kg/day zerumbone compared with their vehicle-treated counterparts (Figure 1B).

### Effects of treatments on renal macrophage infiltration

**Figure 2:**
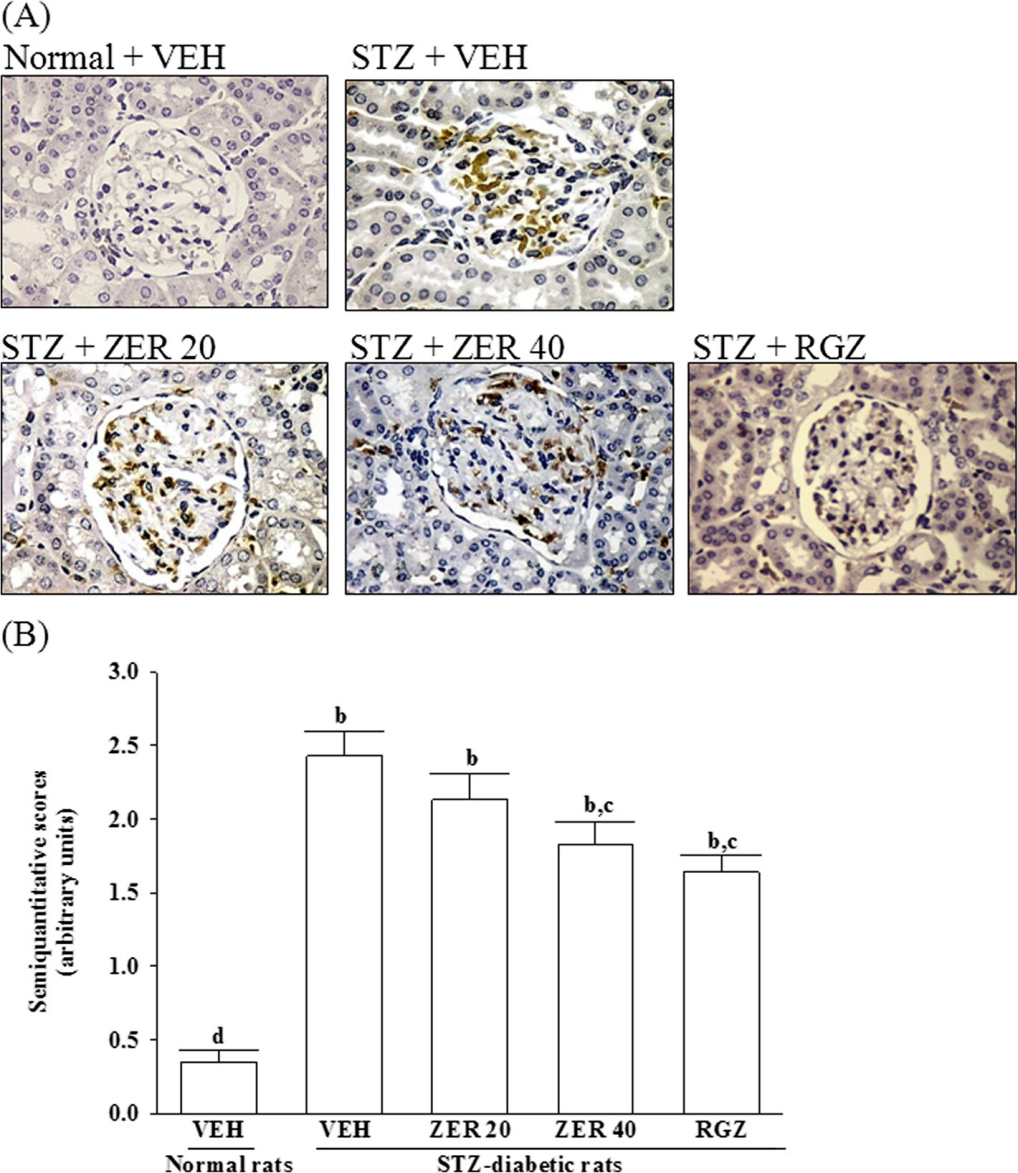
**Effects of treatments on renal macrophage infiltration. (A)** Immunohistochemical staining for macrophage (ED-1-positive) cells in the renal tissues of STZ-diabetic rats treated for eight weeks with zerumbone (ZER) or rosiglitazone (RGZ). STZ-diabetic rats were dosed by oral gavage once daily for eight weeks with 20 mg/kg ZER (STZ + ZER 20), 40 mg/kg ZER (STZ + ZER 40) or 5 mg/kg RGZ (STZ + RGZ). Normal (normal + VEH) or STZ-diabetic rats receiving vehicle treatment (STZ + VEH) were given the same volume of vehicle (VEH) used to dissolve test medications. **(B)** Quantified results are shown for number of macrophages (ED-1-positive cells). Values (mean ± SD) were obtained for each group of 4 animals. ^b^*p* < 0.01 compared to vehicle-treated normal rats. ^c^*p* < 0.05 and ^d^*p* < 0.01 compared to vehicle-treated STZ-diabetic rats, respectively.

Kidneys from control rats showed no significant macrophage infiltration (Figure 2). In contrast, prominent macrophage (ED-1-positive cells) infiltration was evident in the glomeruli of STZ-diabetic rats (Figure 2). Treatment of STZ-diabetic rats with rosiglitazone or 40 mg/kg/day zerumbone for eight weeks caused a 32.5 ± 4.2 and 24.7 ± 3.6% reduction of macrophage influx, respectively, relative to that in their vehicle-treated counterparts (Figure 2).

### Effects of treatments on renal chemokines expression

**Figure 3:**
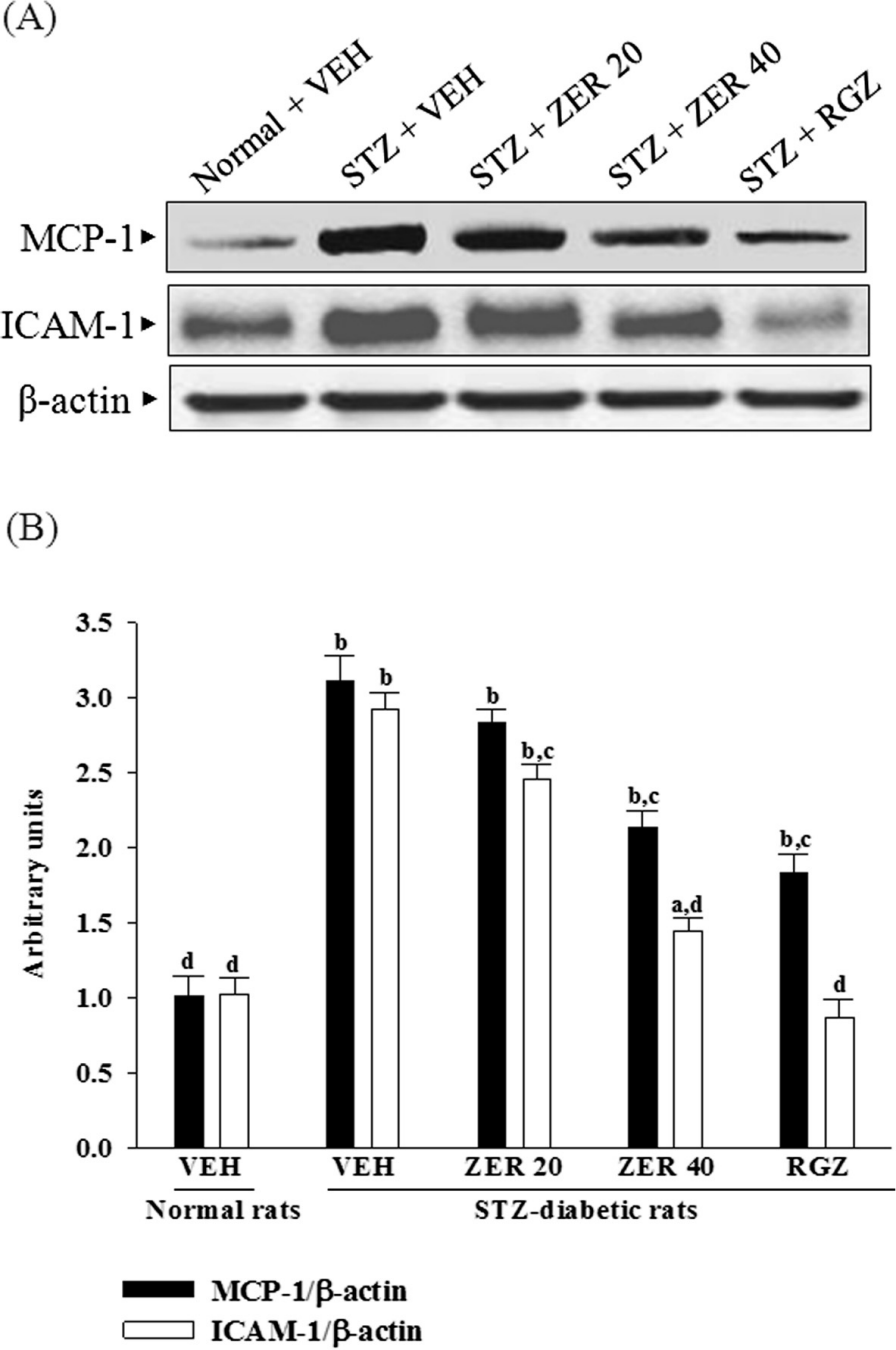
**Effects of treatments on renal chemokines expression. (A)** Representative immunoblots of protein expression of MCP-1 and ICAM-1 in renal tissues of STZ-diabetic rats treated for eight weeks with zerumbone (ZER) or rosiglitazone (RGZ). STZ-diabetic rats were dosed by oral gavage once daily for eight weeks with 20 mg/kg ZER (ZER 20), 40 mg/kg ZER (ZER 40) or 5 mg/kg RGZ (RGZ). Normal (normal + VEH) or STZ-diabetic rats receiving vehicle treatment (STZ + VEH) were administered the same volume of vehicle (VEH) used to dissolve test medications. **(B)** Ratio of MCP-1/β-actin or ICAM-1/β-actin is expressed as the mean with SD (n = 4 per group) in each column. ^a^*p* < 0.01 and ^b^*p* < 0.01 compared to vehicle-treated normal rats. ^c^*p* < 0.05 and ^d^*p* < 0.01 compared to vehicle-treated STZ-diabetic rats, respectively.

The renal MCP-1 and ICAM-1 proteins were 3.0 and 2.9 fold higher in STZ-diabetic rats compared with normal rats, respectively. These increases were ameliorated by 74.2 ± 5.1 and 58.7 ± 3.9%, respectively, after eight weeks of treatment with rosiglitazone (Figure 3). Treatment of STZ-diabetic rats with 40 mg/kg/day zerumbone for eight weeks resulted in a marked 58.8 ± 3.7 and 29.3 ± 2.9% reduction of renal MCP-1 and ICAM-1protein expression, respectively, compared with that in vehicle-treated counterparts (Figure 3).

### Effects of treatments on renal inflammatory cytokines expression

A significant increase of renal TNF-α protein was observed in STZ-diabetic rats when compared with the level in the control rats (Table 2). Treatment for eight weeks with rosiglitazone significantly decreased TNF-α protein levels in the kidneys of STZ-diabetic rats to 43.1 ± 2.4% of their vehicle-counterparts (Table 2). Renal TNF-α protein levels were reduced by 20.7 ± 1.7 and 36.7 ± 2.1% after daily treatment with 20 mg/kg and 40 mg/kg zerumbone, respectively, relative to the level in vehicle-treated STZ-diabetic rats (Table 2).

**Table 2:** Renal levels of cytokines in experimental animals at the end of the eight-week treatment

**Cytokines**	**Normal rats**	**STZ-diabetic rats**			
	**Vehicle**	**Vehicle**	**ZER 20**	**ZER 40**	**RGZ**
TNF-α (pg/mg protein)	72.04 ± 8.26^d^	169.35 ± 9.74^b^	134.30 ± 10.33^b,c^	107.19 ± 8.71^b,d^	96.47 ± 7.36^a,d^
IL-1β (pg/mg protein)	23.37 ± 4.17^d^	62.78 ± 5.07^b^	42.26 ± 2.97^b,c^	33.21 ± 3.81^b,c^	29.88 ± 3.74^b,c^
IL-6 (pg/mg protein)	55.73 ± 8.74^d^	164.27 ± 12.32^b^	130.25 ± 9.23^b,c^	109.17 ± 10.51^b,c^	87.52 ± 5.13^b,c^

STZ-diabetic rats were dosed by oral gavage once per day for eight weeks with 5 mg/kg/day rosiglitazone (RGZ), 20 mg/kg/day zerumbone (ZER 20), 40 mg/kg/day zerumbone (ZER 40). Normal or STZ-diabetic rats receiving vehicle treatment were given the same volume of vehicle (distilled water) used to dissolve test medications. Values (mean ± SD) were obtained for each group of 8 animals.

^a^*p* < 0.05 and ^b^*p* < 0.01 compared to the values of vehicle-treated normal rats.

^c^*p* < 0.05 and ^d^*p* < 0.01 compared to the values of vehicle-treated STZ-diabetic rats, respectively.

The renal concentrations of IL-1β and IL-6 in STZ-diabetic rats were also significantly higher to 2.9 and 2.6 fold, respectively, relative to their vehicle-treated controls. After 8 weeks of rosiglitazone treatment, the higher renal levels of IL-1β and IL-6 were approximately 43.3 ± 1.9 and 46.9 ± 2.2% lower in STZ-diabetic rats compared to their vehicle-counterparts (Table 2). Treatment of STZ-diabetic rats with zerumbone for eight weeks significantly and dose-dependently reduced renal IL-1β and IL-6 levels (Table 2).

### Effects of treatments on renal TGF-β1 and fibronectin protein expression

**Figure 4:**
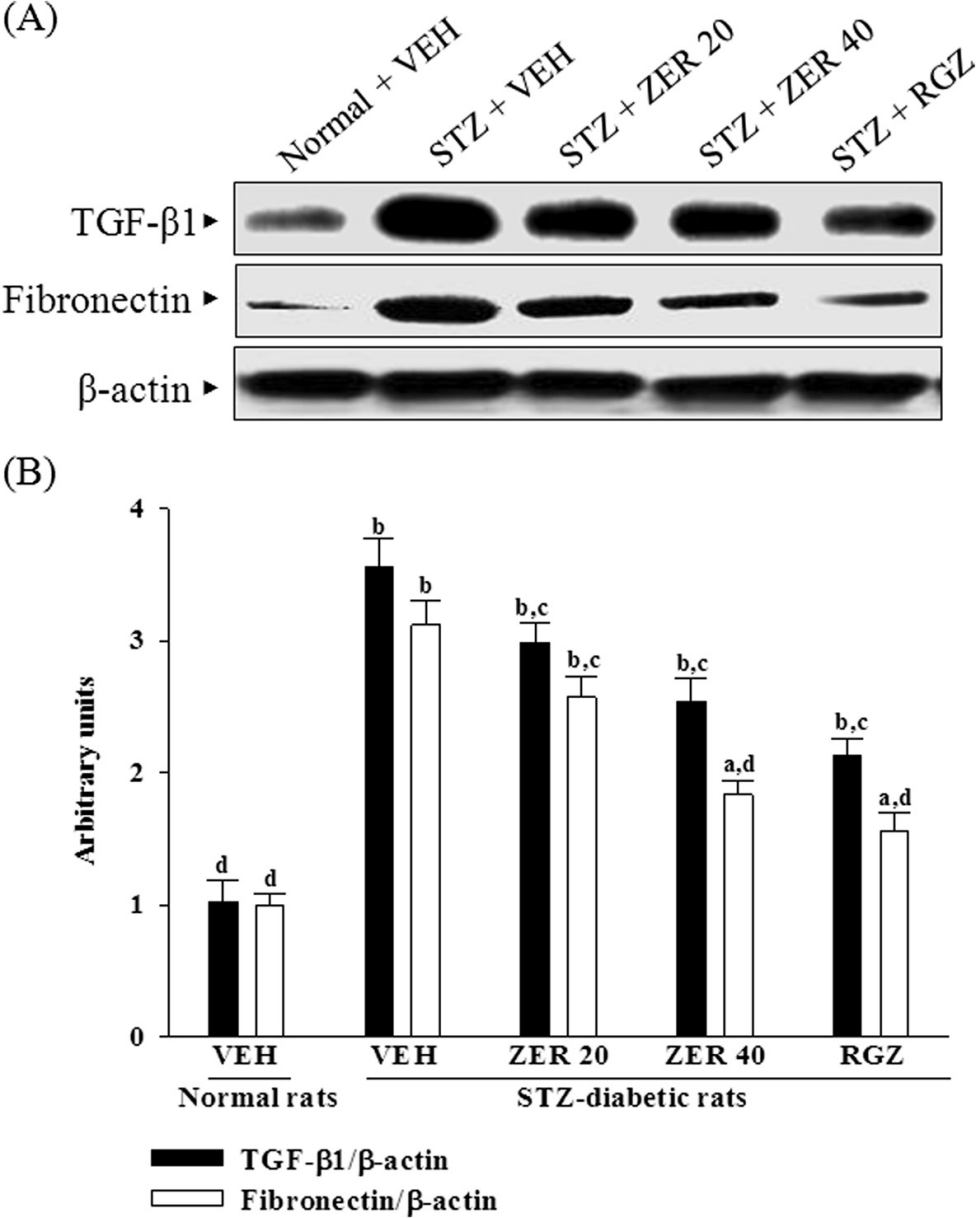
**Effects of treatments on renal TGF-β1 and fibronectin protein expression. (A)** Representative immunoblots of protein expression of TGF-β1 and fibronectin in renal tissues of STZ-diabetic rats treated for eight weeks with zerumbone (ZER) or rosiglitazone (RGZ). STZ-diabetic rats were dosed by oral gavage once daily for eight weeks with 20 mg/kg ZER (ZER 20), 40 mg/kg ZER (ZER 40) or 5 mg/kg RGZ (RGZ). Normal (normal + VEH) or STZ-diabetic rats receiving vehicle treatment (STZ + VEH) were administered the same volume of vehicle (VEH) used to dissolve test medications. **(B)** Ratios of TGF-β1/β-actin and fibronectin/β-actin are expressed as the mean with SD (n = 4 per group) in each column. ^a^*p* < 0.01 and ^b^*p* < 0.01 compared to vehicle-treated normal rats. ^c^*p* < 0.05 and ^d^*p* < 0.01 compared to vehicle-treated STZ-diabetic rats, respectively.

Renal TGF-β1 protein levels were 3.6-fold higher in STZ-diabetic rats relative to normal rats; rosiglitazone treatmet attenuated this increase by 43.7 ± 5.1% (Figure 4). Zerumbone treatments caused dose-dependently downregulation in renal TGF-β1 in STZ-diabetic rats (Figure 4).

STZ treatment significantly increased the fibronectin protein level (by 3.1 fold relative that of vehicle-treated normal rats) in the kidney (Figure 4). The STZ-induced upregulation of fibronectin protein was reduced 17.6 ± 2.3 and 41.3 ± 4.3% relative to that in vehicle-treated STZ-diabetic rats after eight weeks of treatment with 20 and 40 mg/kg/day zerumbone, respectively (Figure 4).

### Effects of treatments on the protein expression and phosphorylation of p38

**Figure 5:**
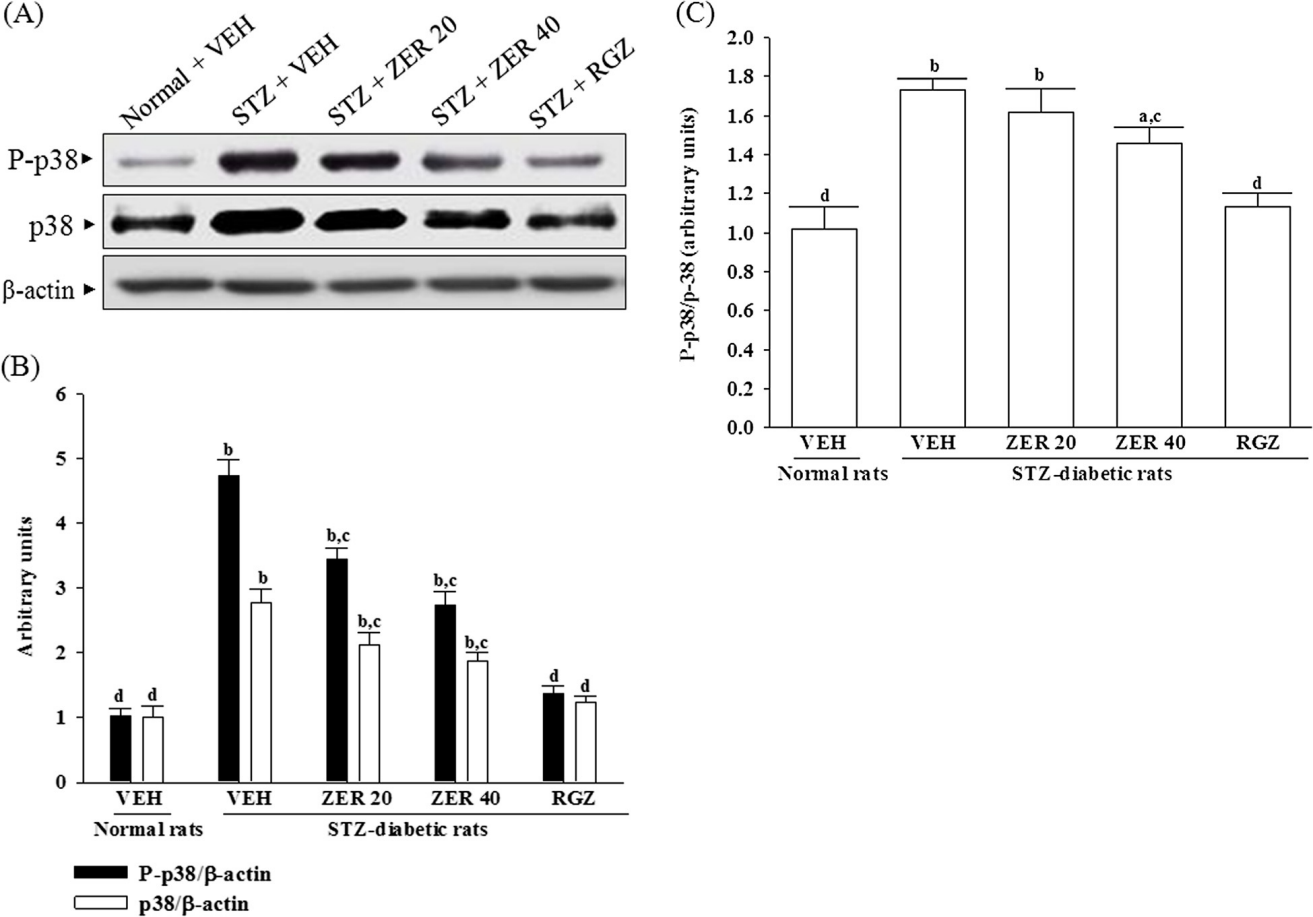
**Effects of treatments on the protein expression and phosphorylation of p38. (A)** Representative immunoblots of protein expression and phosphorylation of p38 in the renal tissues of STZ-diabetic rats for eight weeks with zerumbone (ZER) or rosiglitazone (RGZ). STZ-diabetic rats were dosed by oral gavage once daily for eight weeks with 20 mg/kg ZER (ZER 20), 40 mg/kg ZER (ZER 40) or 5 mg/kg RGZ (RGZ). Normal (normal + VEH) or STZ-diabetic rats receiving vehicle treatment (STZ + VEH) were administered the same volume of vehicle (VEH) used to dissolve test medications. **(B)** Ratios of P-p38/β-actin and p38/β-actin, or **(C)** P-p38/p38 are expressed as the mean with SD (n = 4 per group) in each column. ^a^*p* < 0.01 and ^b^*p* < 0.01 compared to vehicle-treated normal rats. ^c^*p* < 0.05 and ^d^*p* < 0.01 compared to vehicle-treated STZ-diabetic rats, respectively.

The immunoblot results showed that the protein levels and phosphorylation degree of p38 were 2.8 and 4.7 fold higher in kidney of STZ-diabetic rats as compared to the normal group (Figure 5). The STZ also significantly increased the P-p38/p38 ratio (by 1.7 fold relative to those in vehicle-treated normal rats) in kidney of the rats (Figure 5). These STZ-induced up-regulations in protein levels and phosphorylation degree of p38 were reversed in the kidney after 8-week treatment with rosiglitazone by 55.7 ± 4.9 and 70.8 ± 5.4% decreases relative to those in vehicle-treated STZ-diabetic rats (*p* < 0.05, Figure 5). The ratio of P-p38/p38 were reversed in the kidney after 8-week treatment with rosiglitazone by 34.6 ± 2.6% decreases relative to those in vehicle-treated STZ-diabetic rats (*p* < 0.05, Figure 5).

Eight weeks of treatment of STZ-diabetic rats with 20 and 40 mg/kg/day zerumbone decreased p38 protein levels to 76.6 ± 6.1 and 64.7 ± 5.8% of that in vehicle-treated counterparts, respectively (Figure 5). The P-p38 in STZ-diabetic rats were reversed after 8-week treatment with 20 and 40 mg/kg/day zerumbone by 27.6 ± 3.3 and 42.3 ± 4.7% decreases relative to those in their vehicle-treated counterparts (*p* < 0.05, Figure 5). Treatment STZ-diabetic rats with 20 and 40 mg/kg/day zerumbone also significantly down-regulated the ratio of P-p38/p38 in the kidney by 13.6 ± 2.9 and 25.6 ± 3.4% decreases as compared to that in vehicle-treated STZ-diabetic rats (*p* < 0.01, Figure 5).

## Discussion

Analysis of renal biopsies from type 1 and type 2 diabetic patients who develop DN indicate that inflammatory infiltrates are similar in both groups [3], which is consistent with studies in diabetic animal models [19,20]. In the present study, we used the STZ-induced rat DN model to evaluate the potential of zerumbone to inhibit the progression of DN and to investigate the possible underlying mechanism of action.

It is widely known that hyperglycemia can induce microalbuminuria by a hyperfiltration mechanism. Untreated diabetic rats developed severe hyperglycemia with polyuria as a result of osmotic diuresis. Similar to the effects of rosiglitazone, we found that zerumbone administration significantly inhibited the glycosylation of hemoglobin by lowering hyperglycemia in STZ-diabetic rats. The STZ-diabetic rats treated with zerumbone showed an impressive decrease in the amount of proteinuria in parallel with the decrease in urinary volume, probably as a result of the amelioration of hyperglycemia. We also found that the kidney/body weight ratio was reduced by zerumbone after 8 weeks treatment, suggesting that they may reverse kidney hypertrophy in STZ-diabetic rats. Thus, we demonstrated treatment with zerumbone attenuated DN syndrome characterized by proteinuria and the loss of renal function in STZ-diabetic rats. These results suggested that zerumbone might have a beneficial effect on the development and progress of renal injury in the STZ-induced diabetic rats, which was dependent of glycemic control. Currently, the therapy for diabetes aims at maintaining or improving the secretory capacity of the pancreatic β-cell and increasing sensitivity of the target organs such as liver, muscle and adipose tissues, to insulin [21]. In the present study we observed that zerumbone made little effect on the the regulation of pancreatic insulin release. It is obvious that the pancreas might not be the targets of zerumbone bioactivity. It would be of considerable interest to further elucidate the mechanism underlying the plasma glucose lowering action of zerumbone.

Among the many potential pathogenetic mechanisms that are responsible for the development of diabetic kidney disease, an inflammatory mechanism has been suggested to be involved in the development of DN [1]. Macrophages are key inflammatory cells mediating kidney inflammation in experimental and human diabetes. In diabetes, macrophage accumulation and activation are associated with prolonged hyperglycemia, glomerular immune complex deposition, increased chemokine production, and progressive fibrosis [19,20]. Activated macrophages elaborate a host of proinflammatory, profibrotic, and antiangiogenic factors. Using accumulation of ED-1 as a marker of macrophage activation [22], we have demonstrated that increased macrophage activation in the glomeruli of kidney tissue from STZ-diabetic rats is ameliorated by the administration of zerumbone. The renal expression of inflammatory cytokines such as TNF-α, IL-6 and IL-1β were demonstrated to increase in diabetes, contributing to the development of DN [6]. Along with the effects on macrophages, there was a reduction in the upregulated protein expression of TNF-α, IL-6 and IL-1β from kidneys of STZ-diabetic rats receiving zerumbone treatment. Thus, we believe that the anti-inflammatory effects of zerumbone, through the inhibition of macrophage infiltration, might provide a renoprotective effect in the STZ- diabetic model.

ICAM-1 is a known important downstream inflammatory factor whose overexpression promotes inflammatory cells, including mononuclear macrophage infiltration into glomeruli and renal interstitium, as well as accelerates glomerular sclerosis in diabetes [4]. In addition to acting as a chemoattractant cytokine, MCP-1 may be involved in the inflammatory response by activating the macrophages from the circulation to the local kidney and then promote the expression of other proinflammatory cytokines to augment the accumulation of extracellular matrix [4,5]. Consistent with these previous reports, we observed that ICAM-1 and MCP-1 expression were increased in experimental DN and that the increases were attenuated by zerumbone treatment. Therefore, a possible mechanism for preventing the progression of renal disease may involve the effect of zerumbone to attenuate inflammation, by reducing the release of inflammatory mediators and/or inhibiting the expression of adhesion molecules in the diabetic kidney.

The profibrotic factor such as TGF-β1 is recognized as another important factor in the pathogenesis of DN by mediating inflammatory response, which aggravates extracellular matrix accumulation, as well as accelerates glomerularbrosis in diabetes [23]. Thus, the inhibition of TGF-β1 expression benefits the treatment of diabetic kidney disease by alleviating matrix accumulation. Morphologically, the diabetic rats developed glomerular sclerosis with increased PAS-positive extracellular matrix synthesis and increased mesangial matrix area in the glomeruli, however, zerumbone was able to ameliorate the morphological deterioration. Furthermore, fibronectin, a major extracellular matrix protein, was induced in diabetic rats but normalized by zerumbone [24]. Our results also show that, along with the decreases in MCP-1 and ICAM-1 expression, zerumbone treatment significantly decreases TGF-β1 expression. Therefore, we propose the reduced accumulation of glomerular extracellular matrix in zerumbone-treated diabetic rats is a consequence of reduced infiltration of inflammatory cells, in addition to the antifibrotic effect of zerumbone.

**Figure 6:**
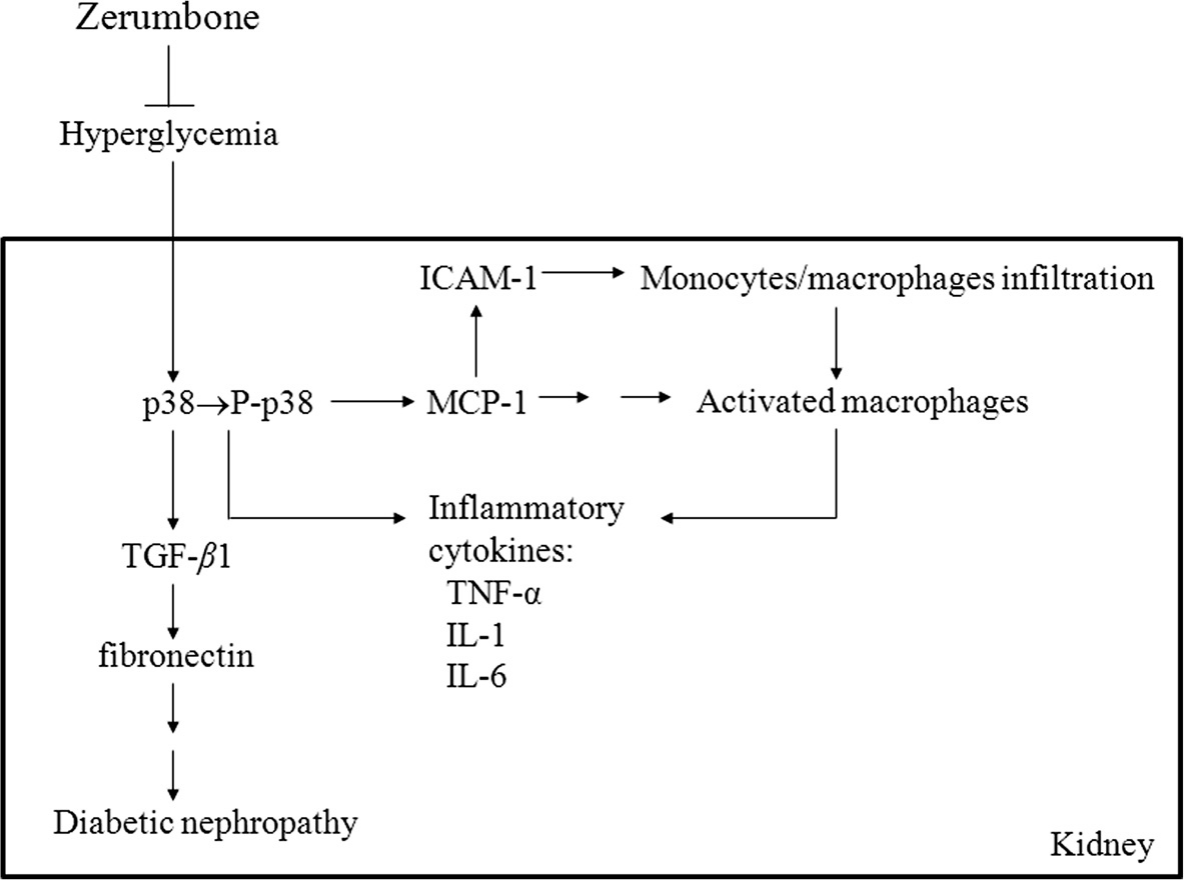
**The possible action mechanisms of zerumbone on the amelioration of DN in STZ-diabetic rats.** Hyperglycemia induced p38 activation to augment the expression of ICAM-1and MCP-1, thereby enhanced the infiltration of monocytes and/or macrophages. The activated macrophages promoted the expression of inflammatory cytokines including TNFα, IL-1 and IL-6. All of the above abnormalities were reversed by zerumbone treatment. Zerumbone also decreased the expression of fibronectin, by reducing the TGF-β1 expression in the diabetic kidney.

In diabetic animal models, p38 activity rapidly increases in glomeruli and tubules after the induction of hyperglycaemia, and is also found in the accumulating kidney interstitial cells associated with advanced nephropathy [12,25]. Increased renal cortical p38 activity in diabetic rats could be attenuated by improved glycemic control [26]. Treatment with zerumbone also reduced the elevated levels of p38 in the kidneys of STZ-diabetic rats, suggesting that the renal protective effect of zerumbone might be also related with the modulation of p38 signal transduction to attenuate hyperglycemia-affected renal dysfunction. We thus concluded that attainment of good glycemic control by zerumbone treatment could abrogate the increased renal p38 pathway activation in diabetic rats and led to minimize risk of DN. A possible mechanism for the renoprotective effect of zerumbone in STZ-diabetic rats is indicated in Figure 6. Although the renoprotective effect of zerumbone is not as effective as that produced by the standard drug rosiglitazone, an agonist of the peroxisome proliferator-activated receptor γ, zerumbone may be a suitable therapeutic adjunct for the patients who are particularly sensitive to the thiazolidinediones-associated side effects [27].

Using a metabolism coefficient of 6.25 to convert the effective daily oral dose of zerumbone for hamsters (40 mg/kg) into a clinical dose, assuming an average adult body weight of 60 kg [28], we estimated a daily oral dose of zerumbone for humans to be approximately 384 mg. However, the placebo controlled human studies are required to find the usability of zerumbone in human DN indications. Also safety testing should be taken with the chronic consumption of large doses of this compound, especially in pregnant women, children, and old people.

## Conclusion

We have shown thatt the beneficial effect of zerumbone in rats with DN is at least in part through antihyperglycemia which was accompanied by inhibition of macrophage infiltration via reducing p38 mediated inflammatory response. Given these promising preclinical findings, we believe that zerumbone might be considered as potential adjuvant entity for DN treatment.

## Competing interests

The authors declare that they have no competing interests.

## Authors’ contributions

TTF contributed to study design, data interpretation and manuscript writing. SSL supervised the work and evaluated the data. CJC performed the experiments and analysis and participated to data interpretation. IML supervised the work, evaluated the data, manuscript writing and corrected the manuscript for publication. All authors contributed to the drafting of the manuscript and agreed on the final version of the manuscript.
